# A Novel Variant in *CLCN7* Regulates the Coupling of Angiogenesis and Osteogenesis

**DOI:** 10.3389/fcell.2020.599826

**Published:** 2020-11-16

**Authors:** Hui Peng, Hong-Bo He, Ting Wen

**Affiliations:** ^1^Department of Endocrinology, Endocrinology Research Center, Xiangya Hospital of Central South University, Changsha, China; ^2^Department of Orthopedic, Xiangya Hospital of Central South University, Changsha, China

**Keywords:** autosomal dominant osteopetrosis type II, *CLCN7*, variant, CD31^hi^Emcn^hi^ vessel formation, bone formation

## Abstract

Autosomal dominant osteopetrosis type II (ADO II), characterized by increased bone mass and density, is caused by mutations in the chloride channel 7 (*CLCN7*) gene. In this study, a novel missense variant in *CLCN7* (c.1678A > G; p.Met560Val) was identified in three symptomatic subjects and one carrier of a Chinese family with ADO II. Notably, bone formation markers, including osteocalcin and total procollagen type N-terminal propeptide, have increased or presented at the upper limit of the normal range in the three patients. Serum factors secreted by osteoclast lineage cells and affecting the CD31^hi^EMCN^hi^ vessel formation, such as tartrate-resistant acid phosphatase 5b, platelet-derived growth factor-BB, vascular endothelial growth factor, and SLIT3, had a higher expression in three ADO II subjects than in 15 healthy age-matched and sex-matched controls. Moreover, the conditioned medium was obtained from preosteoclast induced from the ADO II patients’ peripheral blood mononuclear cells. It was found to promote the CD31^hi^EMCN^hi^ vessel formation of human microvascular endothelial cells and osteogenic differentiation of bone marrow-derived stem cells. Taken together, our finding revealed a novel *CLCN7* variant associated with ADO II and suggested that the sclerotic bone was potentially associated with the increase of the CD31^hi^EMCN^hi^ vessel formation and bone formation.

## Introduction

Autosomal dominant osteopetrosis type II (ADO II; OPTA2, Online Mendelian Inheritance in Man: 166600), also known as “Albers Schönberg disease,” is the most common type of osteopetrosis with an estimated prevalence of 2.5/100,000 (Bénichou et al., 2000; Del Fattore et al., 2008). It is characterized by segmentary osteosclerosis, predominantly involving in the vertebral endplates (“rugger-jersey spine”), iliac wings (“bone within bone” sign), and the skull base. Previous studies revealed that ADO II was a phenotypically heterogeneous disease whose clinical spectrums ranged from unaffected radiography mutation carriers to affected skeletal patients yet asymptomatic to severe manifestations including fracture, osteomyelitis, visual loss, and bone marrow failure (Bénichou et al., 2000; Waguespack et al., 2007).

The bone homeostasis was maintained by balancing osteoclast-mediated resorption and osteoblast-mediated formation (Wang et al., 2018; Hayashi et al., 2019; Rodriguez-Laguna et al., 2019). The disrupted bone mass and structure of osteopetrosis resulted from the impaired osteoclast-mediated bone resorption caused by genetic factors (Tolar et al., 2004; Sobacchi et al., 2013). Bénichou et al. (2001) previously considered that ADO II was genetically homogeneous with a single disease-causing gene on 16p13.3. Accumulated studies showed that roughly 70% of patients harbored heterozygous missense mutations of the *CLCN7* gene in a dominant-negative form (Cleiren et al., 2001). To date, more than 40 mutations in *CLCN7* has been identified in different osteopetrosis families (Leisle et al., 2011; Pang et al., 2016). *CLCN7* (Online Mendelian Inheritance in Man: 602727) is a multi-pass membrane protein that acts as a Cl-/H + exchanger by voltage gate (Leisle et al., 2011). It mainly resides in the late endosomes, lysosomes, and the ruffled membrane of osteoclasts. In osteoclasts, the ruffled border contains Cl-channels that are inserted by exocytotic fusion together with the H + -ATPase to ensure the acidification of extracellular resorption lacuna. Pathogenic mutations in *the CLCN7* gene impairing acidification of extracellular resorption lacuna were assumed to prevent degradation of the calcified and organic matrix of bone (Weinert et al., 2010). Furthermore, some studies suggested that chloride channels could be a drug target for osteopetrosis (Capulli et al., 2015). Mattia et al. demonstrated that *CLCN7* mutant-specific small interfering RNAs silenced dominant-negative *CLCN7* transcripts and subsequently ameliorated the bone phenotype in ADO II (Verkman and Galietta, 2009). Recent research also demonstrated that the specific small interfering RNA therapy normalized extra-skeletal manifestations on the ADO II mouse (Maurizi et al., 2019).

Some studies suggested that *CLCN7* mutations regulate bone formation in patients with osteopetrosis (Barvencik et al., 2014; Coudert et al., 2014). The vessels in the bone niche were reported to play great roles in bone formation (Abarrategi et al., 2018; Hao et al., 2019). A specific endothelium in the murine skeletal system, termed type H, strongly positive for CD31 and endomucin, is crucial in bone formation and regeneration (Kusumbe et al., 2014; Ramasamy et al., 2014; Langen et al., 2017). Accumulated evidence suggests that the type H vessels exert on bone formation in many aspects, ranging from mediating local growth of the vasculature, to providing a necessary microenvironment for perivascular osteoprogenitors, and coupling angiogenesis to osteogenesis (Yang et al., 2017). Previous findings revealed that platelet-derived growth factor-BB (PDGF-BB) secreted by preosteoclast could induce CD31^hi^Emcn^hi^ vessels subtype subsequently prevented bone loss in osteoporosis and bone-loss diseases (Xie et al., 2014). Furthermore, we recently found a mutation in *Reg1cp* involved in high bone mass syndrome through promoting angiogenesis (Yang et al., 2019). However, whether the bone accrual is associated with the coupling between angiogenesis and osteogenesis or not remains unclear.

Here, we ascertained a missense variant in *CLCN7* in a Chinese family. This is the first description genetically confirmed with this heterozygous missense mutation (c.1678A > G; p. Met560Val) responsible for ADO II. Additionally, these results indicated that the mutation might be associated with the dysfunction of the angiogenesis and osteogenesis, which further explained the elevated bone formation markers in this ADO II family.

## Materials and Methods

### Family Collection

**FIGURE 1:**
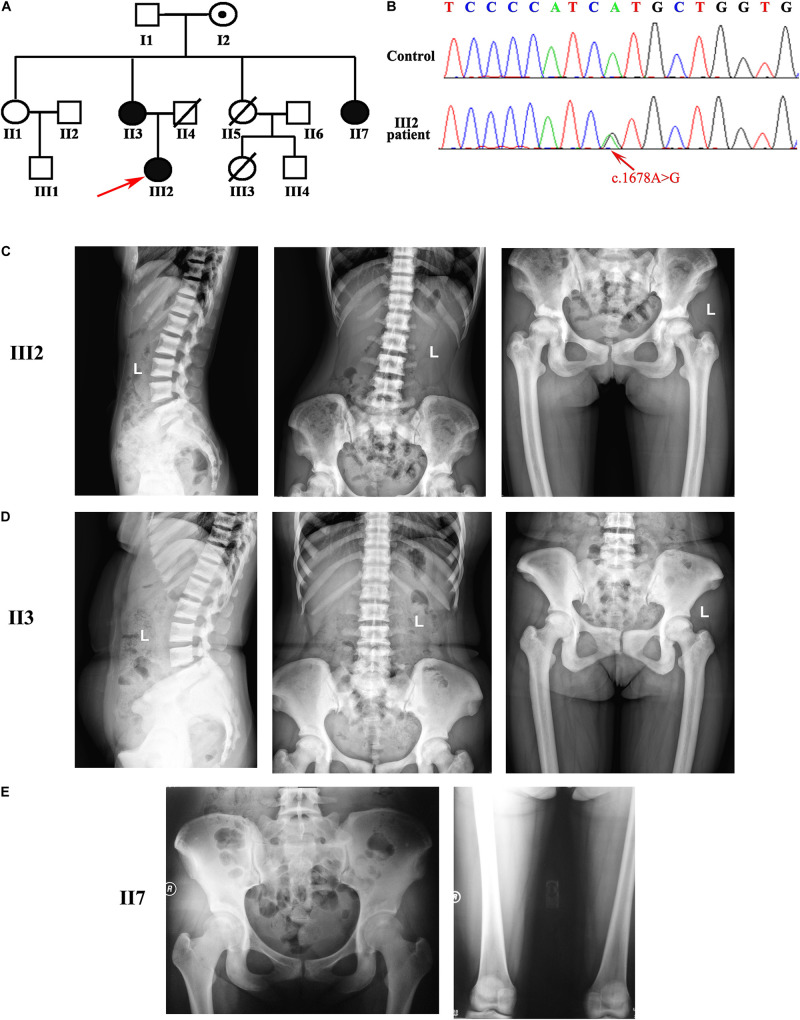
Pedigree of the osteopetrosis family was associated with the mutation in *CLCN7*. **(A)** Pedigree of a Chinese family with osteopetrosis. Squares represent males, and circles represent females. Red arrow indicates the proband in this family. White symbols represent unaffected subjects, and black symbols indicate affected subjects. Symbols with an embedded black dot represent asymptomatic carriers. Symbols with a diagonal line represent deceased subjects. **(B)** Sanger sequence chromatograms from the proband and unaffected II1. c.1678A > G transition is indicated by the red arrow. **(C–E)** Radiographic image reveals osteosclerosis in three affected patients (radiographic images of III2 in the top row, images of II3 in the second row, and images of II7 in the bottom row). Images showed vertebral endplates (“rugger-jersey spine”; III2 and II3) and osteosclerosis of the pelvis and femoral (III2, II3, and II7).

The Chinese family who suffered from ADO II was recruited from Xiangya Hospital Central South University. There were three affected individuals (II3, II7, and III2 in Figure 1A) in this family, and all of the affected subjects were not born of consanguineous parents. The patients underwent a series of clinical evaluation including laboratory and radiography examination in Xiangya Hospital, such as blood route, liver function test, renal function test, serum calcium (Ca), phosphate (P), alkaline phosphatase (ALP), β-Cross Laps of type I collagen containing cross-linked C-telopeptide, osteocalcin (OC), total procollagen type N-terminal propeptide (TPINP), and intact parathyroid hormone (PTH). Additionally, the patients were carried out with bone mineral density of the total body measured by a dual-energy X-ray absorptiometry densitometer and X-ray of bilateral hip joints and lumbar. The study was approved by the Ethics Committee of Xiangya Hospital Central South University. All subjects involved in this study signed informed consent documents before participating in the project.

### Whole-Genome Sequence

Genomic DNA was collected and extracted from peripheral whole blood samples using Qiagen DNA kits. The genomic DNA met the sequencing requirements of the purity (optical density 260/280 > 1.8) and concentration (50 ng/ml) of each sample. Whole-genome sequencing was conducted by Annoroad Gene Technology by using Illumina/TruSeq Nano DNA HT Library Preparation Kit for capture and Illumina sequencing platforms for data generation (150-bp paired-end, 300 cycles). Sequencing was performed with 150-bp paired-end reads on the Illumina NovaSeq 6000 sequencing platform. The mean coverage was 40×.

### Variant Filtering and Analysis

Sequence alignment was performed with the Burrows–Wheeler Aligner mem using human genome assembly hg19 as the reference, then produce called variants with RTG tools Hyplotype Caller. Annotation of gene context was carried out with annovar using Single Nucleotide Polymorphism Database, Refseq gene as the reference.

### Sanger Sequence

The variant was amplified by using DNA polymerase [MIX (GREEN), TSINGKE, China], and the former and reverse primers are CTTGAGGAGTCCACACCCAC and CTGGGAGCAGGTGGATGG, respectively. Forward and reverse sequencing reactions were performed with the Big Dye Terminator Cycle Sequencing Ready Reaction Kit (PE Applied Biosystems), and the products were analyzed on an ABI 3730XL automated sequencer (PE Applied Biosystems).

### Enzyme-Linked Immunosorbent Assay

Serum was obtained from peripheral blood using 4,000 rpm for 5 min. Then, we used the ELISA kits to test the expression of tartrate-resistant acid phosphatase 5b (TRAP-5b), PDGF-BB, vascular endothelial growth factor (VEGF; R&D Systems), and SLIT3 (SED353Mi) in serum according to the manufacturers’ instructions. The ELISA was replicated three times for the affected patients, and the mean values were used.

### Cell Culture

A total of 15 ml of heparin anti-coagulated peripheral blood was obtained from every subject, including all members of this family and healthy age- and sex-matched controls. Then, peripheral blood mononuclear cells (PBMCs) were washed using phosphate-buffered saline twice and cultured in minimum essential medium Eagle-α containing 10% fetal bovine serum, 100 U ml^–1^ penicillin, 100 μg ml^–1^ streptomycin sulfate, and 50 ng ml^–1^ macrophage colony-stimulating factor (M-CSF; R&D Systems Inc.). Non-adherent cells were incubated with M-CSF (50 ng ml^–1^) to obtain pure monocytes and macrophages. Then, PBMCs were cultured in 24-well plates at 1 × 10^5^ cells per well with 50 ng ml^–1^ of M-CSF and 100 ng ml^–1^ receptor activator of nuclear factor-kappa-B ligand. Cells became preosteoclasts after a 3-day culture. We collected serum-containing conditioned medium from the preosteoclasts. After centrifugation (2,500 rpm for 10 min at 4°C), we aliquoted conditioned media and stored them at −80°C. Human BMSCs (HUXMA-0100; Cyagen Biosciences) were cultured in a human mesenchymal stem cell growth medium (HUXMA-90011; Cyagen Biosciences). To induce osteogenic differentiation of BMSCs, BMSCs were cultured in 48-well using the serum-containing conditioned medium. Then, we collected protein to test ALP activity on day 2, RNA on the 3rd day, and performed the ALP staining on day 7. Human microvascular endothelial cells (HMECs) were cultured with MCDB131 medium (Gibco) supplemented 10% fetal bovine serum, 100 U ml^–1^ penicillin, and 100 μg ml^–1^ streptomycin.

### Real-Time Quantitative Polymerase Chain Reaction

Quantitative PCR analysis was performed as described previously (Xiao et al., 2020). Briefly, we extracted RNA from cells using Trizol reagent (Takara). Then, 1,000-ng RNA was reverse-transcribed into first-strand complementary DNA using the Reverse Transcription Kit (Takara). Quantitative PCR was performed using SYBR Green PCR Master Mix (Takara), and messenger RNA expression was normalized to reference genes *GAPDH*.

### Tube Formation Assay

The Matrigel (BD Biosciences) was plated in 48-well plates and then incubated at 37°C for 45 min. HMECs were seeded in 48-well plates at 5 × 10^4^ cells per well on polymerized Matrigel. The cells were cultured in the preosteoclast-conditioned medium collected from patients or control individuals for 4 h incubation at 37°C. Then, the tube formation was observed through microscopy, and the number of tube branches was quantified in four random microscope visual fields of each well.

### Alkaline Phosphatase Activity

On the second day of osteogenic differentiation, the cell lysates were homogenized for ALP activity assay by spectrophotometric measurement of *p*-nitrophenol release using an ALP Assay Kit (P0321S, Beyotime) according to the manufacturer’s instructions.

### Alkaline Phosphatase Staining

The cells were washed using phosphate-buffered saline for three times, followed by 10% paraformaldehyde for 5 min. Then, cells were incubated in ALP incubation buffer (0.2-*g* barbital sodium, 0.4-*g* magnesium sulfate, 0.2-*g* calcium chloride, and 0.3-*g* beta-glycerophosphate) at 37°C for 2 h. Next, cells were washed with 2% calcium chloride and incubated with 2% cobaltous nitrate for 5 min. Finally, cells were incubated in 1:80 ammonium sulfate for 10 s. The ALP staining was observed through a microscope.

### Statistical Analysis

Data were imported into Excel and scaled and normalized to appropriate controls. Unpaired, two-tailed Student’s *t*-tests were performed for the comparisons of two groups. Critical *P*-values were Bonferroni corrected and expressed as follows: *P < 0.05. **P < 0.01.

## Results

### Clinical Characteristics of Patients

A Chinese patient, aged 22 years, was admitted to the Department of Endocrinology with a history of bone pain for 6 years. There was a family history of a similar disease of her mother (II3) and aunt (II7) shown in Figure 1A. All these three patients had a mild phenotype of osteopetrosis only with bone pain on hip joints and back. The proband also had a history of fracture in the hip. On examination, we did not find poor nutritional status, short stature, malformed craniofacial appearance, hepatosplenomegaly, and intellectual disability in the proband and other affected patients in this family.

Laboratory findings of the three patients showed that the hematopoietic function, liver function, kidney function, total serum calcium, and total serum phosphorus were within the normal ranges except for a slight increase of aspartate transaminase of II3 (Table 1). For bone turnover markers, β cross-linked C-telopeptide of type 1 collagen and ALP have not detected any abnormalities in this family (Table 2). However, markers of bone formation in the proband, such as OC and TPINP, were both found at the upper limit of the normal range (Table 2). Moreover, the levels of OC, TPINP, and PTH (parathormone) were found to be elevated in her mother (II3). The level of OC in her aunt (II7) also slightly increased. Thus, we hypothesized that bone formation in these patients had been elevated (Table 2).

**TABLE 1:** Hematopoietic, hepatorenal, and serum electrolytic data of ADO II subjects.

**Parameters**	**III2**	**II3**	**II7**	**Normal range**
WBC (10^9^⋅L^–1^)	4.2	5.3	5.46	3.5–9.5
Hb (g⋅L^–1^)	124	126	126	115–150
RBC (10^12^⋅L^–1^)	4.52	4.34	4.19	3.80–5.10
PLT (10^9^⋅L^–1^)	295	240	146	125–350
ALT (μ/L)	9.3	21.6	20.4	7.0–40.0
AST (μ/L)	32.4	46.5	22.5	13.0–35.0
BUN (mmol/L)	4.58	5.56	6.95	2.60–7.50
Cre (μmol/L)	55	69	53	41.0–111.0
UA (μmol/L)	287.5	289.5	246	155.0–357.0
Na (mmol/L)	140	142.8	142	137.0–147.0
K (mmol/L)	4.54	4.22	3.6	3.50–5.30
Cl (mmol/L)	103.4	107.8	105.6	99.0–110.0
Ca (mmol/L)	2.45	2.28	2.22	2.00–2.60
Mg (mmol/L)	0.91	0.84	0.98	0.66–1.07
P (mmol/L)	1.41	1.45	1.25	0.86–1.78

*WBC, white blood cells; RBC, red blood cells; Hb, hemoglobin; PLT, platelet; ALT, alanine transaminase; AST, aspartate transaminase; BUN, blood urea nitrogen; Cre, creatinine; and UA, uric acid.*

**TABLE 2:** Bone biomarker of ADO II subjects.

	**III2**	**II3**	**II7**	**Normal range**
ALP (μ/L)	55.3	64.5	46.8	45.0–125.0
OC (ng/ml)	20.44	26.79	45	4.11–21.87
TPINP (ng/ml)	58.37	131.4	NA	8.53–64.32
PTH (pg/ml)	44.8	83.8	59.85	15.0–68.3
β-CTX (pg/ml)	290.5	243.5	572	68–680

*ALP, alkaline phosphatase; OC, osteocalcin; TPINP, total procollagen type N-terminal propeptide; PTH, parathormone; β-CTX, β cross-linked C-telopeptide of type 1 collagen; and NA, not available.*

The total bone mineral density values of the three patients at lumbar and hip sites, measured by dual-energy X-ray absorptiometry, are shown in Table 3. The three affected patients were all with regular menstruation. The *Z* values at different sites of the three patients were significantly higher than the those of the sex-matched and age-matched controls (Table 3). Radiological features of the three patients revealed diffused osteosclerosis, predominantly at the vertebral endplates (“rugger-jersey spine”). The superior ramus of the pubis, sacral vertebrae, femoral head, and greater trochanter of femur also presented diffuse osteosclerosis. The boundaries among cancellous bone, cortical bone, and bone medullary disappeared (Figures 1C–E). Based on their clinical and radiological assessment, the family was diagnosed with ADO II.

**TABLE 3:** Bone mass density of the ADO II subjects.

**Subjects**	**Age**	**Total**		**Lumbar**		**Left hip**	
		**Total BMD (g/cm^2^)**	***Z* value**	**Total BMD (g/cm^2^)**	***Z* value**	**Total BMD (g/cm^2^)**	***Z* value**
III2	21	1.611	5.8	2.221	12.1	1.591	6.9
II 3	42	2.063	8.0	2.304	12.7	1.918	9.6
II7	29	1.973	7.6	2.245	11.2	1.855	8.3

*BMD, Bone mineral density.*

### Genetic Analysis With Whole-Genome Sequencing and Identification of the Pathogenic Variant

We performed the whole-genome sequence in the three affected patients. Firstly, we filtered the common variants, which were unlikely to cause the rare heritable disorder (allele frequency greater than 0.1% in the 1000 Genomes database and Exome Variant Server). Then, we excluded the variants with low confidence and the putative impact. Next, we tried to find the shared variant in the three patients. Interestingly, a heterozygous missense variant (c.1678A > G, p.Met560Val) in *CLCN7* segregated with osteopetrosis among three affected patients. We then performed the Sanger sequence in all members of this family and found the variant occurred in three patients and a non-penetrant carrier (I2 in Figure 1A was asymptomatic and did not present abnormal bone phenotype as well as laboratory findings; Figure 1B).

**FIGURE 2:**
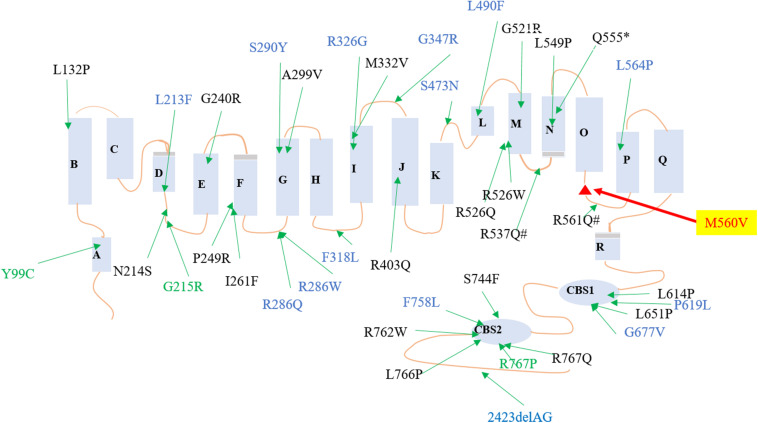
Reported mutations of *CLCN7* in osteopetrosis family. Red mutation was found in this family. Black mutations were reported to cause osteopetrosis autosomal recessive 4, blue mutations were reported to cause ADO II, and green mutations were reported to cause different types of osteopetrosis.

**FIGURE 3:**
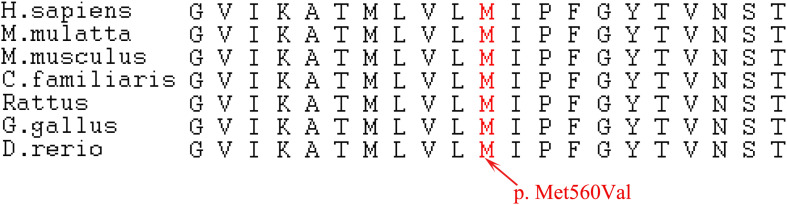
Conservation of the mutated amino acids. Alignment of the *CLCN7* proteins, showing the conservation of the mutated residues among different species. p. Met560Val transition is indicated by the red arrow.

Various mutations in *CLCN7* have been reported to be involved in the pathogenesis of osteopetrosis (Figure 2). In gnomAD database, the PopMax Allele Frequency and allele count of this variant were 0.00000900058 and 1, respectively, (the total Allele number was 245442). Moreover, this variant was not found in East Asian with 17228 Allele number in total. Pro560 in *CLCN7*, which is substituted to a Valine in three patients, is phylogenetically highly conserved among both vertebrates and invertebrates (Figure 3) [Phylop] score of 6.111 and [PhastCons] score of 1. *CLCN7* gene was also predicted to be intolerant whose Residual Variation Intolerance Score is 7.0381% (Table 4). Furthermore, the effect of missense mutation Met560Val in *CLCN7* was predicted by different variant effect prediction software (PolyPhen-2, MutationTaster, CADD, MPC). It suggested that the Met560Val substitution is likely to be deleterious (PolyPhen score of 0.991, probably damaging; MutationTaster score of 0.999, disease-causing; CADD score of 24.3, damaging; MPC score of 1.0788, possibly damaging; Table 4). Based on the genetic analysis, this study revealed that the heterozygous missense mutation was possibly responsible for the ADO II.

**TABLE 4:** Summary of the *CLCN7* mutation in this family with ADO II.

**Variant (Missense)**	**Numbers**		**Rarity in gnomAD**		**Conservation (Extremely conserved)**		**Pathogenic prediction (Damaging)**			
	**Patients**	**Carrier**	**PopMax AF**	**Allele count**	**Phylop score**	**PhastCon score**	**MutationT-aster score**	**MPC**	**CADD score Phred**	**RVIS%**
c.1678A > G p.Met560Val	3	1	9.00058E-06	1	6.111	1	0.999	1.0788	24.3	7.04%

*PopMax AF, Maximum allele frequency in any one population; MPC, mutant prevention concentration; CADD, Combined Annotation Dependent Depletion; and RVIS, Residual Variation Intolerance Score.*

### *CLCN7* Mutation Induces the Type H Vessel Formation and Osteogenic Differentiation *in vitro*

**FIGURE 4:**
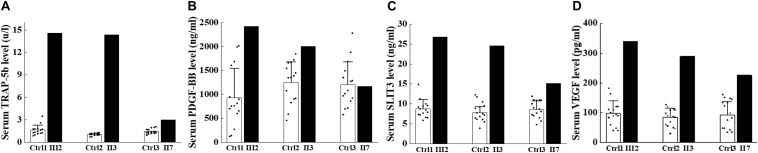
Expression of factors secreted by osteoclast lineage cells elevated in the affected patients. Serum analysis of ADO II subjects and age-matched and sex-matched. **(A)** Expression of serum TRAP-5b. **(B)** Expression of serum PDGF-BB. **(C)** Expression of serum SLIT3. **(D)** Expression of serum VEGF. Number of controls = 15. Data are shown as mean ± SD.

**FIGURE 5:**
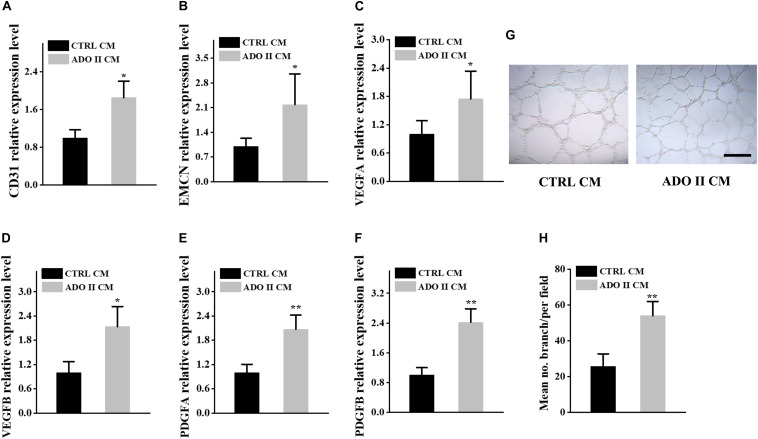
Preosteoclast-conditioned medium from ADO II patients promotes vessel formation *in vitro*. HMECs were treated with a preosteoclast-conditioned medium from ADO II patients or control groups. **(A,B)** qRT–PCR analysis of the relative levels of *CD31* **(A)**, *EMCN* **(B)**, *VEGFA* **(C)**, *VEGFB* **(D)**, *PDGFA* **(E)**, and *PDGFB* **(F)** in HMECs. **(G)** Representative images of tube formation. **(H)** Quantification of tube branch numbers per field. CTRL CM, control conditioned medium; ADO II CM, ADO II conditioned medium. These experiments were replicated three times. Data are shown as mean ± SD. *P < 0.05 and **P < 0.01.

**FIGURE 6:**
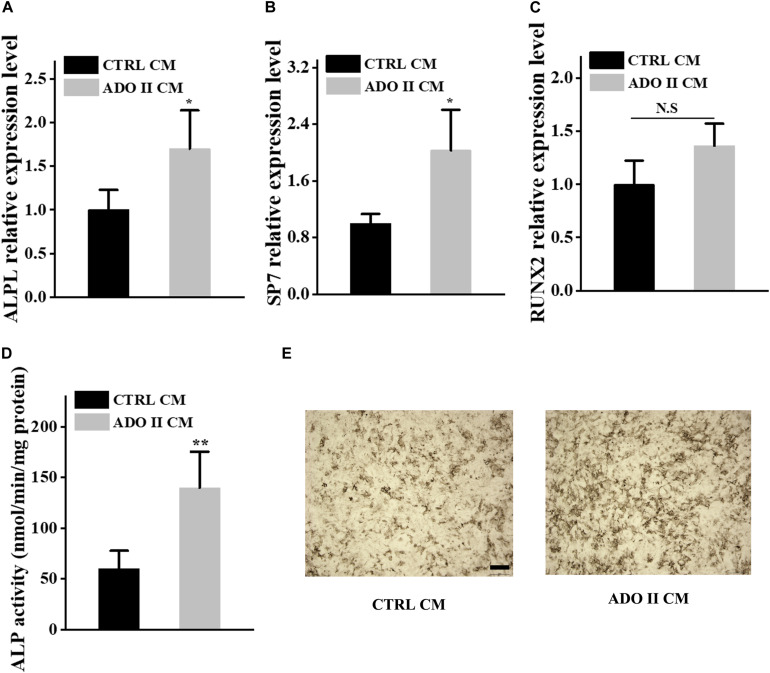
Preosteoclast-conditioned medium from ADO II patients enhances osteoblastic differentiation *in vitro*. BMSCs were treated with a preosteoclast-conditioned medium from ADO II patients or control groups. **(A–C)** qRT–PCR analysis of the relative levels of *ALPL* **(A)**, *SP7* **(B)**, and *RUNX2* **(C)** in BMSCs. **(D)** ALP activity in BMSCs. **(E)** Representative images of ALP staining in BMSCs. Scale bar = 300 μm. These experiments were replicated three times. Data are shown as mean ± SD. *P < 0.05 and **P < 0.01.

The cross-talks between osteoclasts and other cells, including osteoblasts, endothelium cells, have been well established (Phan et al., 2004; Ramasamy et al., 2014; Xie et al., 2014; Yang et al., 2017, 2019; Chen et al., 2018; Li et al., 2019). Additionally, PDGF-BB secreted by preosteoclasts could result in elevated bone mass through inducing angiogenesis during coupling with osteogenesis (Xie et al., 2014). To investigate if the osteosclerotic bone of these patients was associated with the coupling between angiogenesis and osteogenesis, we collected the serum from ADO II individuals and 15 normal controls for further studies. TRAP-5b is well-established to be secreted by osteoclasts, and PDGF-BB, VEGF, and SLIT3 can also be secreted by osteoclasts/preosteoclasts, which were reported to be involved in the type H formation (Bollerslev et al., 2013; Sivaraj and Adams, 2016; Kim et al., 2018; Peng et al., 2020). Thus, we selected them to see if they have been altered in ADO II patients. Surprisingly, the level of TRAP-5b, PDGF-BB, VEGF, and SLIT3 showed a remarkable increase in ADO II patients compared with the 15 control groups with matched age and sex (Figures 4A–D). To further confirm the possible effect of type H vessels in this kindred, we purified the PBMCs from the patients and control normal subjects. Then, the collected preosteoclast condition medium from osteoclastic-induced PBMCs has been used to culture the HMECs. As expected, the expression of *CD31*, *EMCN*, and vessel growth factors, including *VEGFA*, *VEGFB*, *PDGFA*, and *PDGFB*, has increased in the cells treated with preosteoclast-conditioned medium from ADO II cells compared with the cells from control individuals (Figures 5A–F). Moreover, the tube formation also has been promoted by the ADO II-conditioned medium (Figures 5G,H). Even the expression of *RUNX2* was not altered by the conditioned medium from patients; other osteogenic markers in hBMSCs, such as *ALPL* and *SP7*, elevated (Figures 6A–C). Consistent with these results, both ALP activity and ALP staining showed a significant difference between the two groups (Figures 6D,E). Together, these data suggested that the *CLCN7* mutation might cause the bone accrual partially because of the elevated coupling of bone formation with type H formation.

## Discussion

Osteopetrosis is a rare genetic condition of increased bone mass and density generally due to a defect of bone resorption. The common complications are confined to the skeleton, including bone fracture, bone pain, osteomyelitis, and rare bone marrow failure (Bénichou et al., 2000). Bone pain on the back was described in three affected patients and fracture on the hip of the proband. Their prominent radiographic signatures of vertebral endplates (“rugger-jersey spine”) and diffused bone sclerosis were found in three affected patients.

The mutation analysis in this family was in agreement with the clinical diagnosis of ADO-II. Previous studies that reported several mutations in *CLCN7* could result in severe recessive, dominant, and intermediate osteopetrosis (Frattini et al., 2003). Currently, genetic and molecular diagnoses were available for patients with ADO II, especially to the missense mutations in the *CLCN7* gene (Bollerslev et al., 2013). Above 70% of patients harbored heterozygous dominant-negative mutations of the *CLCN7* gene (Cleiren et al., 2001). These *CLCN7*-related mutations are usually located in different structures of its protein, such as intramembrane α helices, extracellular loops, Cl-binding sites, and CBS domains (Pang et al., 2016; Jentsch and Pusch, 2018). We identified a heterozygous variant p.Met560Val located in the loop between helix N and helix O in three patients and an unaffected carrier. There was only one allele of the identified mutation in gnomAD and was not presented in East Asian populations. Additionally, Steven et al. have estimated the penetrance of ADO II to be approximately 66% (62 clinically affected individuals/94 subjects with the gene mutation), which means among the individuals with *CLCN7* mutations, one third were asymptomatic gene carriers without osteopetrosis-related signs in clinical, biochemical, and radiological results (Waguespack et al., 2003). Thus, the carrier did not present the related osteosclerotic bone associated with the incomplete penetrance. The phenotypical and genetic heterogeneity and incomplete penetrance remain unclear in skeletal dysplasia (Alam et al., 2017; Chen et al., 2020). It is likely to be related to the environmental factors, methylated gene modification, the complex interaction between other genes and *CLCN7*, and even some unidentified loci (Zhang et al., 2009; Leisle et al., 2011; Boudin et al., 2016), which need more further studies to elucidate.

The previous investigation indicated that ADO was one of the osteoclast-rich forms of osteopetrosis (Bollerslev et al., 2013). The bone biopsies from patients with ADO showed increased size and number of osteoclasts (Bollerslev et al., 1993). Chen et al. revealed that c.1856C > T mutation in *CLCN7* identified in an ADO II case resulted in enhanced but hypofunctional osteoclastogenesis (Chen et al., 2016). Besides, previous studies showed that the serum TRAP-5b was significantly elevated in osteoclast-rich ADO II mice and patients (Chen et al., 2016; Alam et al., 2017). Consistent with these studies, the elevated serum TRAP-5b was also found in these patients with ADO II (Alatalo et al., 2004). The prominently elevated biochemical measurements of TRAP-5b suggested that the variant affected the function of osteoclast and indicated the molecular feature to be osteoclast-rich osteopetrosis in this family.

Osteopetrosis is a clinically heterogenetic disorder. In this Chinese family with the *CLCN7* variant, the serum bone formation markers (OC, TPINP, and PTH) were at the upper limit range in the proband. They also elevated in the proband’s mother (II3), and the level of OC increased remarkably in the aunt (II7). The variable level of bone formation markers in the three affected patients might be associated with the characterization of clinical heterogeneity or some factors, such as age, lifestyle, and other environmental stimulus (Lundberg et al., 2018; Chevalier et al., 2020). Taken together, the upregulated or upper limit range of serum bone formation markers implied that the bone formation increased. It is extensively reported that the coupling of angiogenesis with osteogenesis plays a great role in bone modeling and remodeling (Phan et al., 2004; Ramasamy et al., 2014; Xie et al., 2014; Yang et al., 2017, 2019; Chen et al., 2018; Li et al., 2018). Previous findings revealed that PDGF-BB secreted by preosteoclasts could stimulate angiogenesis during coupling with osteogenesis (Xie et al., 2014). Furthermore, we demonstrated that *Reg1cp* mutation potentially caused high bone mass syndrome by regulating the coupling of angiogenesis with osteogenesis (Yang et al., 2019). Other studies revealed that the secret factors, including VEGF and SLIT3 by osteoclast lineage cells, could regulate type H formation during bone regeneration (Xu et al., 2018; Peng et al., 2020). Based on these findings, we investigated if this *CLCN7* mutation contributed to the increased bone mass by regulating the process of angiogenesis and osteogenesis. Intriguingly, we found that the levels of PDGF-BB, VEGF, and SLIT3 were increased in ADO II individuals. Of note, the conditioned medium from PBMCs of ADO II patients promoted vessel formation of HMECs as well as osteogenic differentiation of hBMSCs. These results gave some new insights into the pathogenesis in this family.

In this study, we identified a novel missense variant (p.Met560Val) in *CLCN7* through whole-genome sequencing and detected it in all affected family members by Sanger sequence. The rarity, conservation, and pathogenic predication all supported that this *CLCN7* mutation might be a disease-causing one. Furthermore, the *in vitro* experiments showed that the variant promoted the vessel formation and osteogenic differentiation in this family. To conclude, we presented a novel heterozygous mutation (p.Met560Val) in *CLCN7* segregated with ADO II in this Chinese family and revealed that it might be associated with the increased type H vessel formation and bone formation.

## Data Availability Statement

The whole-genome sequencing data were deposited on the Genbank (Sequence Read Archive) database with the accession no. PRJNA669897.

## Ethics Statement

The studies involving human participants were reviewed and approved by Ethics Committee of the Xiangya Hospital of Central South University. The patients/participants provided their written informed consent to participate in this study. Written informed consent was obtained from the individual(s) for the publication of any potentially identifiable images or data included in this article.

## Author Contributions

HP designed the experiments, performed whole-genome sequencing analysis, carried out most of the experiments, and drafted the manuscript. TW and H-BH recruited the patients. TW supervised the experiments, analyzed results, and proofread the manuscript. All authors were involved in the final approval of the submitted version.

## Conflict of Interest

The authors declare that the research was conducted in the absence of any commercial or financial relationships that could be construed as a potential conflict of interest.
