# Corrigendum: A novel variant in *CLCN7* regulates the coupling of angiogenesis and osteogenesis

**DOI:** 10.3389/fcell.2025.1616778

**Published:** 2025-05-21

**Authors:** Hui Peng, Hong-Bo He, Ting Wen

**Affiliations:** ^1^ Department of Endocrinology, Endocrinology Research Center, Xiangya Hospital of Central South University, Changsha, China; ^2^ Department of Orthopedic, Xiangya Hospital of Central South University, Changsha, China

**Keywords:** autosomal dominant osteopetrosis type II, *CLCN7*, variant, CD31^hi^Emcn^hi^ vessel formation, bone formation

In the published article, there was an error in Figure 6E as published. ALP staining images in Figure 6E were redundantly used. The corrected Figure 6E and its caption appear below.

**FIGURE 6:**
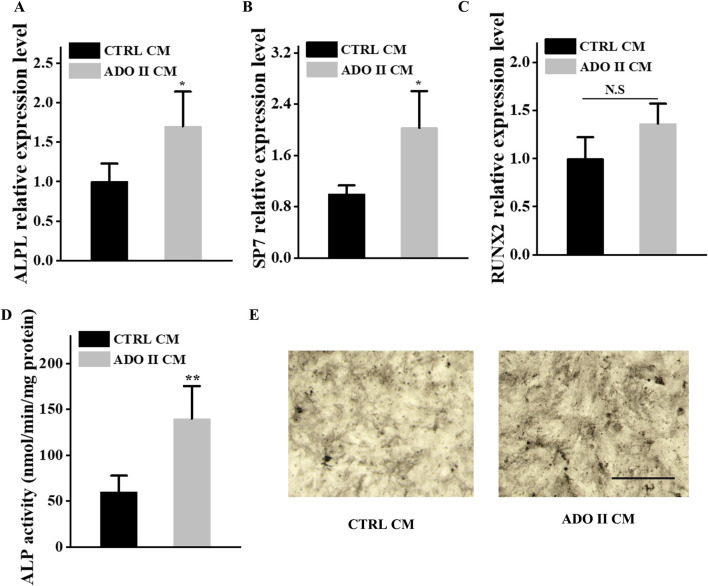
Preosteoclast-conditioned medium from ADO II patients enhances osteoblastic differentiation *in vitro*. BMSCs were treated with a preosteoclast-conditioned medium from ADO II patients or control groups. **(A–C)** qRT–PCR analysis of the relative levels of ALPL **(A)**, SP7 **(B)**, and RUNX2 **(C)** in BMSCs. **(D)** ALP activity in BMSCs. **(E)** Representative images of ALP staining in BMSCs. Scale bar = 300 µm. These experiments were replicated three times. Data are shown as mean ± SD. *P < 0.05 and **P < 0.01.

The authors apologize for this error and state that this does not change the scientific conclusions of the article in any way. The original article has been updated.

